# Impact of semaphorin, Sema3F, on the gene transcription and protein expression of CREB and its binding protein CREBBP in primary hippocampal neurons of rats

**DOI:** 10.1515/biol-2025-1202

**Published:** 2025-12-31

**Authors:** Ting Lv, Xiao-Yang Liu, Jun-Xian Fu, Zhi-Dong Qiao, Guang-Lu Yang

**Affiliations:** Department of Pediatrics, Erdos Central Hospital, Ordos, 017010, China; Department of Pediatrics, Inner Mongolia Autonomous Region Maternal and Child Health Care Hospital, Hohhot, 010010, China; Department of Pediatrics, The Affiliated Hospital of Inner Mongolia Medical University, Hohhot, 010050, China

**Keywords:** CREB, CREBBP, primary hippocampal neuronal system of rats, SD rat, Sema3F

## Abstract

The temporal effects of Semaphorin 3F (Sema3F), a secreted signaling protein, on the transcription and protein expression of cAMP response element-binding protein (CREB) and CREB-binding protein (CREBBP) were evaluated in primary hippocampal neurons derived from Sprague-Dawley rats, with the aim of elucidating their potential involvement in axonal development. An *in vitro* model was employed, in which neurons were treated with 10 ng/ml Sema3F, and outcomes were compared relative to fetal bovine serum controls at 0, 5, 15, and 30-min intervals. Quantitative real-time polymerase chain reaction (PCR) and Western blot analysis demonstrated dynamic regulation in mRNA and protein levels of CREB and CREBBP. A decrease in expression was observed at 5 min post-treatment, followed by a marked increase peaking at 15 min, with continued elevation observed at 30 min. These temporal expression patterns indicate that Sema3F rapidly modulates the expression of CREB and CREBBP, implicating these factors in the regulation of transient transcriptional processes associated with axonal growth and plasticity.

## 1 Introduction

Epilepsy is among the most prevalent and disabling chronic neurological disorders globally, affecting an estimated 60 million individuals annually and contributing to approximately 125,000 deaths each year [1]. The condition is characterized by abnormal electrical discharges within the brain, which manifest as seizures or present with abnormal behaviors, altered sensations, and, in some instances, loss of consciousness. These manifestations can lead to a wide range of neurological, cognitive, psychological, and social consequences. Individuals with epilepsy face a threefold increased risk of premature mortality compared to the general population, contributing substantially to the global burden of disease [2].

Current evidence from both clinical and preclinical studies indicates that the hippocampus frequently serves as a focal point for seizure activity. In epilepsy, the hippocampus exhibits structural alterations, including mossy fiber sprouting (MFS), changes in the morphology and density of dendritic branches and spines, and aberrant patterns of neurogenesis [2]. Consequently, efforts to identify the sources of epileptogenesis and develop strategies to inhibit abnormal neuronal electrical activity remain a major focus of contemporary research. Among these structural changes, MFS has emerged as one of the most extensively studied axonal networks implicated in seizure initiation, particularly in relation to the dynamics of axonal growth cone movement in surviving granule cells. Emerging evidence also implicates members of the semaphorin (Sema) family as critical regulators of axonal pathfinding and sprouting in the context of epilepsy [3].

Semaphorins, initially described as collapse-inducing factors, comprise a gene family encoding guidance molecules involved in embryonic nervous system development. Subsequent studies have identified them as a class of signaling proteins. These membrane-binding proteins are critical in neural development, where they contribute not only to axonal growth cone guidance but also to the regulation of neuronal proliferation and migration, synaptogenesis, synaptic function, and dendritic morphology [4], [5], [6]. Among the class 3 semaphorins, Semaphorin 3F (Sema3F) plays a prominent role in modulating axonogenesis and axonal growth, and has been strongly associated with MFS in pathophysiology of epilepsy [4], [5], [6]. Sema3F has been shown to suppress MFS by influencing synaptic transmission in granule cells of the dentate gyrus and pyramidal neurons of the CA1 region in the hippocampus [7]. However, the molecular mechanisms underlying this inhibitory effect remain incompletely understood.

The cyclic adenosine monophosphate (cAMP) response element-binding protein (CREB) was initially characterized as a transcription factor associated with the somatostatin gene promoter, where it exhibited bidirectional and intact expression. The *CREB* gene is located on chromosome 2q33.3 and is expressed in the nuclei of all nucleated cells. The *CREBBP* gene (OMIM #600140), which encodes the CREB-binding protein (CBP), is located on chromosome 16p13.3 and spans approximately 155 kb, comprising 31 exons in the coding region [8], 9]. CBP functions as a large, multifunctional transcriptional coactivator. Together with its vertebrate homolog p300, CBP is expressed across a wide range of cell types, including hematopoietic, germ, neuronal, muscle, epithelial cells, hepatic, pulmonary, osteoblastic, and pancreatic cells. CBP and p300 represent two of the most extensively studied and functionally complex transcriptional coregulators.

At the cellular level, CBP regulates key processes such as proliferation, differentiation, and apoptosis by integrating intracellular signaling cascades and facilitating transcription of genes involved in these pathways. At the molecular level, CBP possesses intrinsic histone acetyltransferase activity and has been shown to interact with more than 400 nucleoproteins, thereby mediating transcriptional activation at multiple promoter sites and exerting broad regulatory effects [10], 11].

Previous research has demonstrated that reduced CBP expression in the dorsal hippocampus is associated with impaired memory and synaptic plasticity in 12-month-old THY-Tau22 mice, highlighting CBP’s involvement in hippocampal function and its potential contribution to cognitive processes [12]. The interaction between CREB and CBP has been shown to initiate the transcription and translation of CREB target genes, facilitating neuronal growth, proliferation, differentiation, and maturation, as well as regulating the cell cycle through the CREB phosphorylation signaling pathway [13].

Furthermore, CREB regulates gene transcription involved in modulating cell excitability, neuronal migration, and synaptic plasticity, processes which are fundamental to memory formation and maintenance. CREB activity contributes to the formation of spatial and long-term memory and supports neuronal survival [14], 15].

Multiple studies have demonstrated that Sema3F inhibits MFS by inhibiting neuronal synaptic transmission, thereby reducing the incidence of epilepsy. However, the specific molecular mechanisms through which Sema3F inhibits MFS remain unknown. The present study was designed to investigate the hypothesis that Sema3F may attenuate epileptogenic processes by modulating signaling pathways involving CREB and CREBBP.

## 2 Materials and methods

### 2.1 Laboratory animals

Neonatal Sprague-Dawley (SD) rats, aged 24 h, were obtained from the Laboratory Animal Center of Inner Mongolia Medical University.

**Ethical approval:** The research related to animal use has been complied with all the relevant national regulations and institutional policies for the care and use of animals, and has been approved by the Ethics Committee of the Affiliated Hospital of Inner Mongolia Medical University.

### 2.2 Experimental reagents

The following reagents and materials were used in the experimental procedures: Dulbecco’s Modified Eagle Medium (DMEM; Gibco, USA), poly-l-lysine (Sigma, USA), 0.25 % Trypsin-EDTA (Gibco, USA), Neurobasal Medium (Gibco, USA), B-27 Supplement (Gibco, USA), fetal bovine serum (Gibco, USA), horse serum (Gibco, USA), purified Sema3F protein (R&D Systems, USA), TRIzol Reagent (Tiangen Biotechnology, China), chloroform (Sinopharm Chemical Reagent Co., Ltd., China), isopropanol (Sinopharm Chemical Reagent Co., Ltd., China), HyPure™ molecular biology grade water (HyClone), RevertAid First Strand cDNA Synthesis Kit (Thermo Fisher Scientific), FastStart Universal SYBR Green Master (Rox) (Roche), primers (Tianyi Huiyuan), 5× protein loading buffer (Beijing Solarbio), phosphorylated protease inhibitor (Servicebio), bicinchoninic acid (BCA) protein quantitative detection kit (Servicebio), ammonium persulfate (APS; Shanghai Sinopharm, China), high-efficiency RIPA lysate (Beijing Solarbio), protease inhibitor (Beyotime), Tris base (Beijing Solarbio), glycine (Beijing Solarbio), sodium dodecyl sulfate (SDS; Beijing Solarbio), methanol (Sinopharm), Tween-20 (Solarbio), enhanced chemiluminescence (ECL) substrate (Bio-Rad), Kodak medical X-ray film (Kodak), protein molecular weight marker (TAKARA), goat anti-rabbit IgG (H + L) and goat anti-mouse IgG (H + L) secondary antibodies (Servicebio), among others.

### 2.3 Experimental equipment

The following equipment was utilized in the study: sterile cell culture studio, ultra-clean bench, TDZ4A-WS low-speed automatic balancing centrifuge, SX-700 autoclave, inverted phase-contrast microscope and associated software (Leica, Germany), constant temperature water bath, real-time PCR system, and a microplate reader (BioTek, USA). Additional instrumentation included a protein electrophoresis unit, transfer apparatus, horizontal shaker, chemiluminescence imaging system, electrically heated ventilated drying oven, and refrigeration units maintained at 4 °C, −20 °C, and −80 °C. Additional consumables included a 37 °C CO_2_ incubator (5 % CO_2_), Petri dishes, disposable micropipette tips (different specifications), syringe filters, 200-mesh cell strainers, absorbent paper, aluminum foil, and other standard laboratory supplies.

### 2.4 Culture and grouping of primary hippocampal neurons isolated from 24-h-old rats

Primary hippocampal neurons were isolated from 24-hour-old SD rats and cultured in Neurobasal medium (Invitrogen) supplemented with 10 % bovine serum albumin (BSA), 2 % B27 supplement, and 1 % glutamine, in accordance with previously described protocols [16]. The *in vitro* neuronal model was divided into an experimental group and a control group. The experimental group received Sema3F at a final concentration of 10 ng/mL, while the control group was treated with fetal bovine serum at an equivalent concentration. Samples from both groups were collected at four time points: 0 min, 5 min, 15 min, and 30 min post-treatment.

### 2.5 Real-time PCR

Total RNA was extracted from each experimental group using a commercially available RNA isolation kit, following the manufacturer’s protocol. Reverse transcription was performed using RNase-free, moist heat-sterilized pipette tips and microcentrifuge tubes, followed by quantitative real-time PCR to assess the expression levels of target genes [17]. Primer sequences are provided in Table 1. Relative gene expression was calculated using the 2^−ΔΔCT^ method.

**Table 1:** Primer sequences used for real-time PCR amplification of CREB, CREBBP, and GAPDH.

Primer name	Sequence (5′–3′)
R-CREB-F	GACGGAGGAGCTTGTACCAC
R-CREB-R	TGGCATGGATACCTGGGCTA
R-CREBBP-F	CAAGCGAAACCAACAAACCATC
R-CREBBP-R	GAAGTGGCATTCTGTTGCCC
R-GAPDH-F	AGACAGCCGCATCTTCTTGT
R-GAPDH-R	CTTGCCGTGGGTAGAGTCAT

### 2.6 Western blot analysis

Cells from each group were harvested and lysed using ice-cold RIPA buffer. Lysates were centrifuged at 12,000 rpm for 30 min, and the supernatant was collected. Protein concentrations were determined using a BCA protein assay. Equal amounts of total protein were separated via SDS-PAGE and transferred to polyvinylidene difluoride (PVDF) membranes. Membranes were blocked with 5 % fat-free milk for 2 h at room temperature and subsequently incubated overnight at 4 °C with primary antibodies. Following incubation, membranes were treated with horseradish peroxidase-conjugated goat anti-rabbit or goat anti-mouse secondary antibodies for 2 h at room temperature. Protein expression levels were quantified by measuring band intensities using Image J image processing software. Each group included three biological replicates (*n* = 3/group).

### 2.7 Statistical analysis

All statistical analyses were performed using SPSS version 27.0. Measurement data conformed to a normal distribution and are expressed as mean ± standard deviation (x̄ ± s). Differences across multiple time points were assessed using repeated measures analysis of variance (ANOVA), followed by the least significant difference (LSD) t-test for post hoc pairwise comparisons. A P-value of less than 0.05 was considered to indicate statistical significance.

## 3 Results

### 3.1 CREB and CREBBP mRNA expression in primary hippocampal neurons

**Figure 1:**
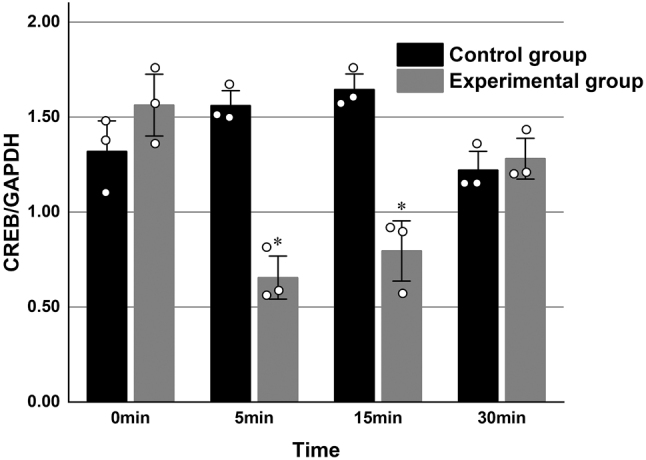
Relative mRNA expression levels of CREB in primary rat hippocampal neurons in the experimental and control groups at identical time points, with GAPDH used as the endogenous reference gene.(*P < 0.05 compared with the experimental group at 0 min; *n* = 3).

**Figure 2:**
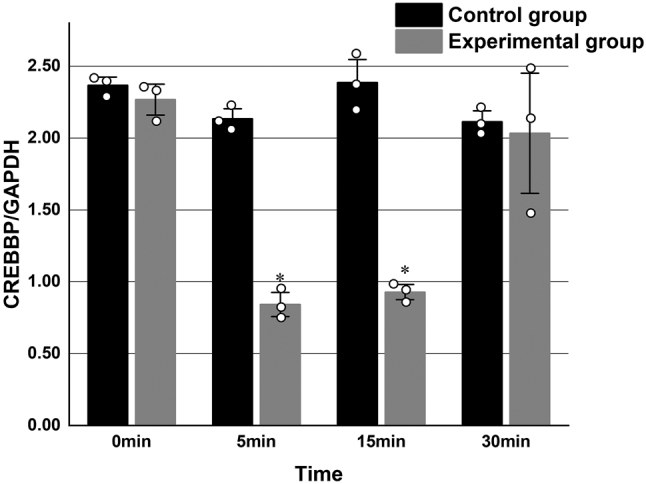
Relative mRNA expression levels of CREBBP in primary rat hippocampal neurons in the experimental and control groups at identical time points, normalized to GAPDH expression. (*P < 0.05 compared with the experimental group at 0 min; *n* = 3).

Quantitative real-time PCR was performed to assess the mRNA expression levels of *CREB* and *CREBBP* in both experimental and control groups at four time points: 0 min, 5 min, 15 min, and 30 min. Each experimental condition was conducted in triplicate and repeated three times. In the experimental group, mRNA expression levels of *CREB* and *CREBBP* demonstrated a significant initial decline followed by a time-dependent increase compared to the control group (*P* < 0.05) (Table 2, Figures 1 and 2).

**Table 2:** Relative mRNA expression levels of CREB and CREBBP in primary rat hippocampal neurons (x ‾ ± s).

		Interaction time				*F*	*P*
		0 min	5 min	15 min	30 min		
CREB	Experimental group	1.47 ± 0.12	0.65 ± 0.13^a^	0.79 ± 0.19^a^	1.28 ± 0.31	82.089	*P < 0.05*
	Control group	1.41 ± 0.19	1.56 ± 0.09	1.64 ± 0.09	1.22 ± 0.12	5.380	0.136
CREBBP	Experimental group	2.27 ± 0.13	0.84 ± 0.10^a^	0.93 ± 0.07^a^	2.03 ± 0.51	21.83	*P* < 0.05
	Control group	2.37 ± 0.07	2.14 ± 0.09	2.39 ± 0.20	2.11 ± 0.09	4.60	0.15

Compared with the experimental group at 0 min, ^a^ *P* < 0.05, *n* = 3; F indicates the variance ratio.

### 3.2 CREB and CREBBP protein expression in primary hippocampal neurons

**Figure 3:**
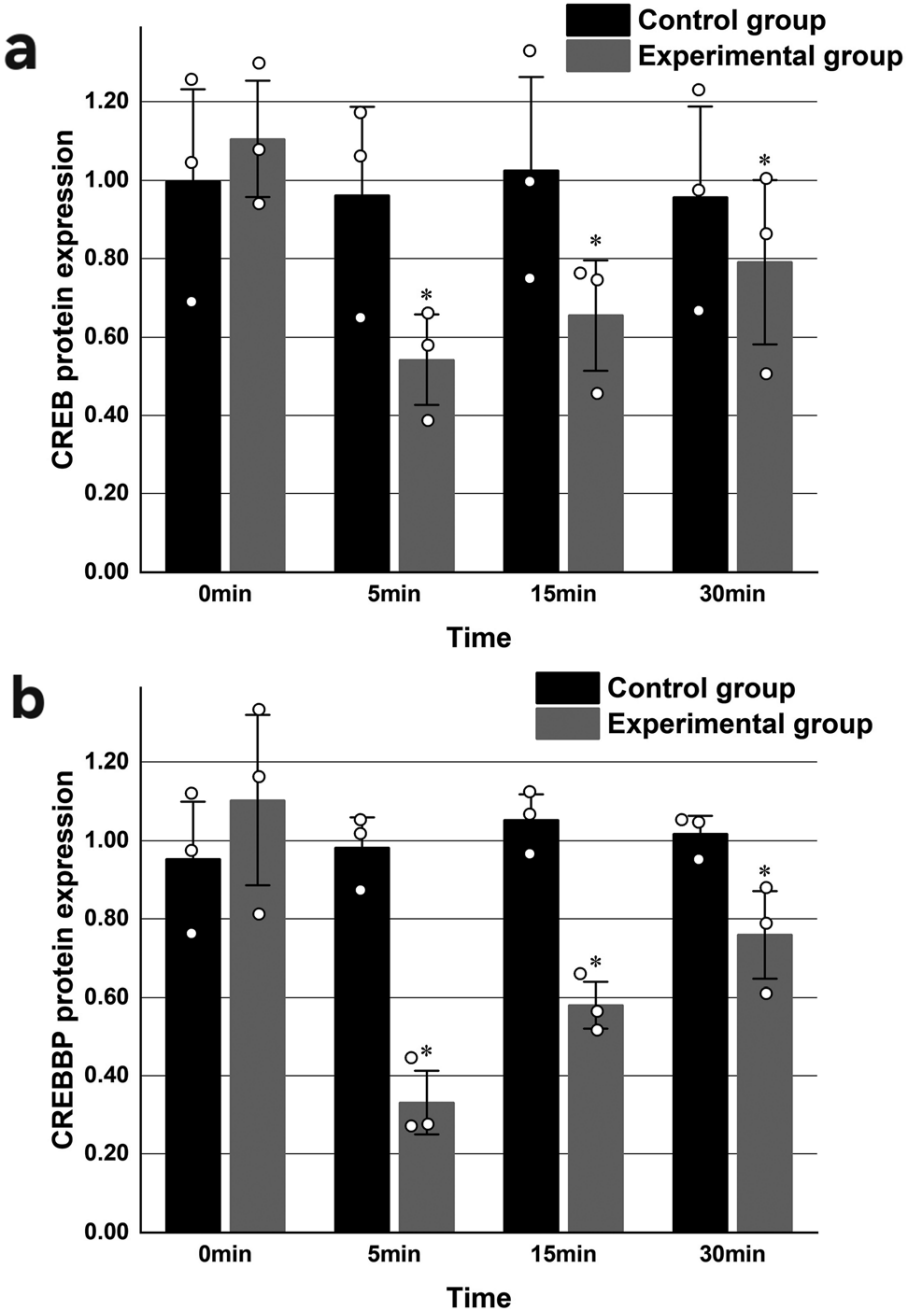
Protein expression levels of CREB and CREBBP in primary hippocampal neurons in the experimental and control groups. GAPDH was used as the loading control. a. Representative Western blot images of CREB and CREBBP expression. b. Quantification of CREB protein expression across time points. c. Quantification of CREBBP protein expression in the experimental and control groups across time point. (*P < 0.05 compared with the experimental group at 0 min; *n* = 3).

Western blot analysis was conducted to evaluate CREB and CREBBP protein expression in samples collected from both the experimental and control groups at 0, 5, 15, and 30 min. Each experiment was repeated three times. In the experimental group, protein expression levels of CREB and CREBBP exhibited a significant initial decrease at 5 min, followed by a progressive increase at subsequent time points, compared to the control group (*P* < 0.05) (Table 3, Figure 3).

**Table 3:** Protein expression levels of CREB and CREBBP in primary rat hippocampal neurons (x ‾ ± s).

		Interaction time				*F*	*P*
		0 min	5 min	15 min	30 min		
CREB	Experimental group	1.11 ± 0.18	0.54 ± 0.14^a^	0.65 ± 0.17^a^	0.79 ± 0.25^a^	26.321	*P* < 0.05
	Control group	2.37 ± 0.07	2.14 ± 0.09	2.39 ± 0.20	2.11 ± 0.09	4.60	0.15
CREBBP	Experimental group	1.10 ± 0.27	0.33 ± 0.10^a^	0.58 ± 0.07^a^	0.76 ± 0.14^a^	33.97	*P* < 0.05
	Control group	0.95 ± 1.8	0.98 ± 0.10	1.06 ± 0.08	1.02 ± 0.06	1.69	0.32

Compared with the experimental group at 0 min, ^a^ *P* < 0.05, *n* = 3; F indicates the variance ratio.

CREB and CREBBBP are transcriptional regulators known to be expressed in nucleated cells. In this study, primary hippocampal neurons derived from postnatal rats were cultured *in vitro*. An inverted phase-contrast microscope was used to monitor neuronal morphology and growth. By the third day of culture, axons exhibited maximal thickness, neuronal growth was most robust, and neurite outgrowths had established interconnected networks. Subsequently, a collapse of the axonal growth cone was observed with prolonged culture. Based on these observations, neurons cultured for three days were selected for subsequent experimentation.

Previous research has shown that axonal growth cone collapse in hippocampal neurons occurs in a dose-dependent manner in response to Sema3F, with the most pronounced effect observed at a concentration of 10 ng/mL [13]. Therefore, 10 ng/mL Sema3F was selected as the final concentration for the current experimental protocol to ensure consistency and optimize the reliability of the findings.

## 4 Discussion

The findings of this study demonstrated that treatment with Sema3F resulted in the modulation of both CREB and CREBBP expression levels in rat hippocampal neurons, with a significant decrease observed at 5 min, followed by a progressive increase at 15 min and 30 min. These results suggested that Sema3F influenced primary hippocampal neurons in rats, potentially modulating neural development and migration through signaling pathways involving CREB and CREBBP.

It is postulated that through modulation of these pathways, Sema3F may inhibit axonal growth and development, thereby inhibiting MFS and reducing susceptibility to seizure activity. Supporting evidence from a study investigating the antiepileptic properties of a water extract of *Lilii Bulbus* (WELB) demonstrated that WELB regulated the expression of CREB, a downstream effector in the Sema3F signaling cascade. The observed temporal pattern of CREB expression observed in that study was consistent with changes in mossy fiber outgrowth, further suggesting that the Sema3F-CREB/CREBBP pathway may play a critical role in the underlying mechanisms underlying epileptogenesis [18].

A review of the literature indicates that CREB phosphorylation primarily occurs via the PKA/CREB/BNDF signaling pathway, which has been associated with the pathogenesis of various neurological disorders, including Alzheimer’s disease [19], [20], [21]. Activation of the CREB-BDNF signaling pathway promotes hippocampal neurogenesis, thereby mitigating the cognitive dysfunction [22], [23], [24], [25]. This signaling axis also appears to contribute to the pathophysiology of Parkinson’s disease by protecting hippocampal neurons from inflammation and oxidative stress-mediated death [26]. CREB has been implicated in modulating synaptic plasticity and is strongly associated with neuroprotective functions, neuronal migration, as well as the regulation of learning and memory. In a study conducted by Ryuta et al. (2016), optogenetic techniques were employed in primary granule cells expressing photoactivated adenylate cyclase (PAC). Under blue light irradiation, PAC produced cAMP, which led to abnormal projection of mossy fiber axons into the inner molecular layer [27], an event indicative of MFS, thereby implicating CREB in the regulation of MFS.

The *CREBBP* gene encodes CBP, a transcriptional coactivator that functions as a molecular scaffold by interacting with a wide range of proteins and facilitating the interpretation of intracellular signals [10]. CBP and its structurally and functionally related homolog, p300, constitute the p300/CBP coactivator family [28], which plays essential and distinct roles *in vivo*. These include regulation of neurogenesis and subsequent neuronal differentiation [28], 29], migration of neuronal cells [18], 30], DNA damage response and repair of both single- and double-strand breaks [31], 32], upregulation of complement activation [33], participation in memory process [34], regulation of long-term memory and circadian rhythms [35], and possessing carcinogenic properties [27], 36], 37]. Additionally, CBP has been implicated in reducing levels of inflammatory markers in models of inflammatory lung injury [38]. It plays a central role in chromatin remodeling and is involved in a variety of biological and pathological processes, including histone acetylation, negative regulation of transcription, and the modulation of signaling pathways involving transforming growth factors *β* receptors, hypoxia responses, and broader intracellular signal transduction mechanisms [39], 40].

Evidence from the literature indicates that cognitive impairment associated with hippocampal injury is mediated, in part, by CREB signaling within the hippocampus. This signaling pathway has been implicated in the regulation of dendritic spine morphology and synaptic remodeling, processes essential for cognitive function [41], 42].

From a clinical perspective, mutations in the *CREBBP* gene have been predominantly associated with Rubinstein-Taybi syndrome, a rare inherited neurodevelopmental disorder characterized by epilepsy and intellectual disability [43]. Previous studies have demonstrated that CBP regulates the development of *γ*-aminobutyric acid neurons *in vivo*. Given that GABAergic neurons function as inhibitory neurons within the central nervous system, their role has been strongly linked to paroxysmal focal seizures (PFS) and the pathophysiology of epilepsy [11], 12].

Alejandro Medrano-Fernández and colleagues reported that CBP is expressed in neural precursors cells within the embryonic medial ganglionic elevation (MGE), a region responsible for generating the majority of cortical interneurons. Using primary cultures derived from MGE precursor cells, CBP was shown to play a critical role in the regulation of interneuron development *in vivo*. Reduced CBP expression was associated with impaired differentiation of forebrain interneurons, potentially contributing to the pathogenesis of epilepsy observed in patients with Rubinstein-Taybi syndrome (RTS) [11], 12], 44]. These findings suggest that CBP may inhibit the GABA signaling pathway, leading to abnormal production of *γ*-aminobutyric acidergic neurons, which leads to the predominance of excitatory neurons and epilepsy.

In a related study, Ryuta et al. (2016) used optogenetic methods to photoactivate primary cultures of granulosa cells expressing adenylate cyclase (PAC). Upon blue light stimulation, PAC-generated cAMP, facilitated CBP formation, which in turn induced aberrant mossy fiber axonal projections into the inner molecular layer, thereby promoting MFS [27].

CBP has been demonstrated to promote mossy fiber sprouting. In the present study, both mRNA and protein expression levels of CREBBP were found to be reduced at 5 and 15 min following the administration of Sema3F, compared to baseline levels at 0 min. This finding indicates that Sema3F suppressed the expression of CREBBP. When considered alongside findings from previous investigations, these results support the hypothesis that downregulation of CREBBP expression may contribute to the inhibition of mossy fiber sprouting.

In prior studies involving pediatric populations with RTS, reduced CBP expression was reported to result in decreased *γ*-aminobutyric acid levels, followed by the onset of seizures. Several possible explanations may account for this apparent discrepancy: (1) CBP’s promotion of GABA expression may predominantly occur during early neurodevelopment, particularly within the embryonic neural layers, while its functional role in later stages of neuronal maturation remains unclear; and (2) *CREBBP* gene mutations implicated in RTS may result in functional alterations that affect other signaling pathways in which CBP is involved, although the specific nature and direction of these mutations were not detailed in the referenced studies.

A previous study reported that during the DNA damage response, junction mediating and regulating protein (JMY), originally characterized through its interaction with the p300/CBP family of transcriptional coactivators, demonstrated increased nuclear localization. This nuclear translocation enhanced p53 activity while attenuating JMY’s role in regulating cell motility, thereby positioning JMY as a cofactor that modulates p53 activity under cellular stress conditions. In addition to its interaction with p300/CBP, JMY was shown to associate with multiple sequence-specific transcription factors, including hypoxia-inducible factor-1 (HIF-1), which plays a critical role in maintaining oxygen homeostasis. [45], [46], [47], [48]. Upon accumulation, HIF-1α translocated to the nucleus and dimerized with HIF-1β. The HIF-1α/β complex recruited transcriptional coactivators such as p300/CBP and bound to hypoxia response elements (HRE), activating the transcription of hypoxia-regulated genes, such as vascular endothelial growth factor (VEGF) [49]. These findings indicate that p300/CBP functions as a transcriptional coactivator for various sequence-specific factors, including p53, HIF-1α, and VEGF [50].

Previous experimental data demonstrated that p53 overexpression attenuated Sema3F-induced axonal growth cone collapse in cultured primary rat hippocampal neurons [16]. Furthermore, Sema3F has been shown to inhibit VEGF expression in a time-dependent manner, as evidenced by reduced VEGF mRNA and protein levels following Sema3F administration at 0, 5, 15, and 30 min. In alignment with these observations, the current findings demonstrated that Sema3F reduced CREBBP expression to a certain extent, further supporting the hypothesis that Sema3F may exert regulatory effects on pathways involving p53 and its associated transcriptional coactivators.

In conclusion, the findings of this study provide additional insight into the role of *CREBBP* in the pathogenesis of epilepsy. Early exposure to Sema3F appeared to suppress mossy fiber outgrowth and may attenuate epileptogenic processes, potentially through downregulation of CREBBP expression and concomitant upregulation of *γ*-aminobutyric acidergic neurons involved in GABA signaling pathways. However, it remains unclear whether the inhibitory effect of Sema3F on CREBBP expression influences VEGF levels and whether these regulatory interactions are mediated through the HIF-1 signaling pathway. Further studies are warranted to elucidate through further investigation into the mechanistic links among Sema3F, CREBBP, and downstream transcriptional networks in the context of epileptogenesis.

Although this study provides novel insights into the acute transcriptional regulation mediated by Semaphorin, the limited sample size (*n* = 3 per group) posed a constrain on broader generalizability. This limitation reflects the inherent technical challenges of primary neuronal culture, where biological variability necessitates stringent quality control, thereby reducing the number of viable replicates. Future research should aim to include larger sample sizes and incorporate *in vivo* validation to strengthen the reliability and translational relevance of these findings. Additionally, while the present study clarified the temporal regulation of CREB and CREBBP by Sema3F, the mechanistic involvement of Sema3F receptors, such as Neuropilin-2 and PlexinA3, as well as the dependency on CREB signaling, remains to be fully established. These aspects will form the focus of subsequent investigations.

## 5 Conclusions

This study demonstrated that CREB and CREBBP contribute to the growth and development of neuronal axons, with evidence suggesting a potential regulatory role of the PKA/CREB/BNDF signaling pathway in these processes.
